# A Quantitative and Dynamic Model for Plant Stem Cell Regulation

**DOI:** 10.1371/journal.pone.0003553

**Published:** 2008-10-29

**Authors:** Florian Geier, Jan U. Lohmann, Moritz Gerstung, Annette T. Maier, Jens Timmer, Christian Fleck

**Affiliations:** 1 Department of Mathematics and Physics, University of Freiburg, Freiburg, Germany; 2 Department of Biology, University of Freiburg, Freiburg, Germany; 3 Department of Molecular Biology, Max Plank Institute for Developmental Biology, AG J. Lohmann, Tübingen, Germany; 4 University of Heidelberg, Heidelberg, Germany; 5 Department of Biosystems Science and Engineering, D-BSSE, ETH Zürich, Basel, Switzerland; 6 Freiburg Institute for Advanced Studies, Freiburg, Germany; 7 Center for Biological Systems Analysis, Freiburg, Germany; University of Glasgow, United Kingdom

## Abstract

Plants maintain pools of totipotent stem cells throughout their entire life. These stem cells are embedded within specialized tissues called meristems, which form the growing points of the organism. The shoot apical meristem of the reference plant *Arabidopsis thaliana* is subdivided into several distinct domains, which execute diverse biological functions, such as tissue organization, cell-proliferation and differentiation. The number of cells required for growth and organ formation changes over the course of a plants life, while the structure of the meristem remains remarkably constant. Thus, regulatory systems must be in place, which allow for an adaptation of cell proliferation within the shoot apical meristem, while maintaining the organization at the tissue level. To advance our understanding of this dynamic tissue behavior, we measured domain sizes as well as cell division rates of the shoot apical meristem under various environmental conditions, which cause adaptations in meristem size. Based on our results we developed a mathematical model to explain the observed changes by a cell pool size dependent regulation of cell proliferation and differentiation, which is able to correctly predict *CLV3* and *WUS* over-expression phenotypes. While the model shows stem cell homeostasis under constant growth conditions, it predicts a variation in stem cell number under changing conditions. Consistent with our experimental data this behavior is correlated with variations in cell proliferation. Therefore, we investigate different signaling mechanisms, which could stabilize stem cell number despite variations in cell proliferation. Our results shed light onto the dynamic constraints of stem cell pool maintenance in the shoot apical meristem of *Arabidopsis* in different environmental conditions and developmental states.

## Introduction

**Figure 1:**
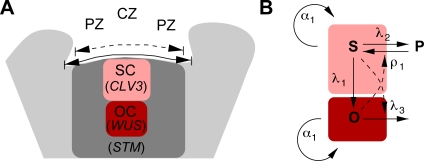
Basic layout of the shoot apical meristem and our model. A) Schematic representation of the SAM. The central zone (CZ) is located in the center of the shoot apex and contains the stem cells (SCs). *CLV3* expression marks the SC domain. Directly below the SC domain is the organizing center (OC), which is defined by the expression of *WUS*. Lateral to the CZ is the peripheral zone (PZ), which consists of rapidly proliferating cells. *STM* expression in the three outer cell layers partially correlates with the PZ. The surface width of the *STM* expression domain (dashed line) as well as the surface distance between opposing primordia (solid line) is indicated. B) Schematic representation of the stem cell pool model. Cells of the SC pool (*S*) and the OC (*O*) proliferate with rate α_1_. SCs differentiate into cells of the OC with rate *λ*_1_ and into cells of the proliferation zone P with rate *λ*_2_. Cells of the proliferation zone can re-specify into SCs with a rate *ρ*_1_. This rate depends on signals from the OC as indicated by the dotted line. Cells of the OC terminally differentiate with a rate *λ*_3_, which depends on the SC pool.

The stem cell (SC) niche in the shoot apical meristem (SAM) of *Arabidopsis* is composed of three functionally distinct zones [1]–[3]. The central zone (CZ), comprising the center of the upper three cell layers, is home to the stem cells (SCs), which divide slowly. Cells that are displaced laterally into the peripheral zone (PZ) remain undifferentiated, but divide more rapidly, before they are incorporated into organ primordia, which are located at the flanks of the SAM. Cells of the organizing center (OC) located below the CZ divide very slowly and are the source of signals that specify SC identity in the CZ and thus set up a functional meristem (see Figure 1A). Despite the fact that the demand for cells varies strongly, the structure of the SAM is remarkably constant over a wide range of environmental conditions and developmental stages. In principle, this variation of cell supply could be achieved by two alternative mechanisms. The size of the SAM could be adapted and thus indirectly lead to a larger cell output rate proportional to the increase in meristem size. Alternatively, the size of the meristem could remain the same while only the cell output rate increases. This latter mechanism requires a shift in the balance of cell proliferation and cell differentiation in the SAM. It is currently unclear, which of these alternative mechanisms operate in the SAM.

It was suggested that non-cell autonomous signaling between the stem cell pool and the OC is responsible for a homeostasis of SC number [4]–[6]. Fundamental to this mechanism is the negative feedback regulation between the homeodomain transcription factor WUSCHEL (WUS) and the short secreted peptide CLAVATA3 (CLV3). *WUS* is expressed in the OC and is essential for the maintenance of SC fate and expression of *CLV3* [5]. CLV3 in turn is secreted by SCs and acts as a non-cell autonomous signal to repress *WUS* expression in the OC via a complex signaling pathway [4], [5], [7]. Additionally, recent experiments showed the possibility of re-specification of peripheral cells into SCs opening another possibility for the regulation of the stem cell pool size [8]. This study also suggested that cell re-specification is regulated by the OC as it is accompanied by an expansion of the *WUS* expression domain. However, how these mechanisms could modulate the overall cell output rate of the SAM under varying conditions is unclear.

Previous modeling approaches of the shoot apex have mainly focused on the question of pattern formation by means of auxin signaling [9]–[12]. Furthermore, Jönsson et al. have used a reaction diffusion model in order to explain the re-formation of the *WUS* expression domain in the SAM after laser ablation of the CZ [13]. How the domain and thus cell pool structure of the SAM is regulated by changes in cell behavior, such as differentiation and proliferation has not been addressed by mathematical modeling so far.

In this study we address the question of SAM regulation quantitatively by combining experimentation and mathematical modeling using data derived from three experimental conditions. We determined the sizes of the SC domain, the OC and the PZ and measured cell proliferation rates in these domains. Our data revealed that the size of the SC pool as well as the size of the OC is correlated with the cell proliferation rate and is not invariant in different environmental conditions. We used this information to develop a mathematical model of the CZ, which can explain variations in cell pool sizes by a balance of cell proliferation and differentiation rates. The model allows us to estimate the unobserved cell differentiation rates of the different cell pools and sheds light on the contribution of SC proliferation towards overall cell production of the SAM. We show that a model based on the well-established negative feedback between SC and OC domains is sufficient to explain *CLV3* and *WUS* over-expression phenotypes. However, the model does not allow a SC homeostasis under variable cell proliferation rates. By examining two possible feedback mechanisms, which both act to buffer the size of the SC pool despite large changes in cell proliferation rates, we identify functional constrains between an adaptation of the SAM to external cues and SC homeostasis.

## Results

### Experimental analysis of cell behavior in the shoot apical meristem

**Figure 2:**
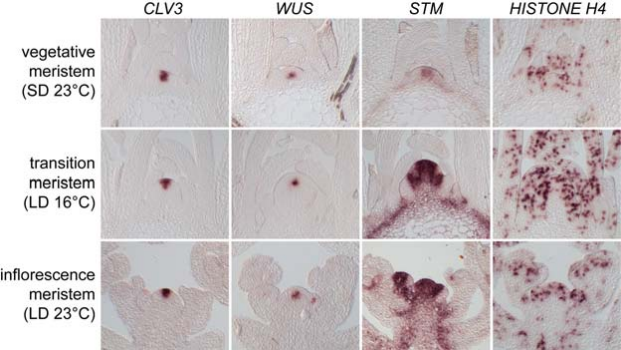
Expression of marker genes. Representative slides of *in situ* hybridizations using *CLV3*, *WUS*, *STM* and *HISTONE H4* RNA probes on tissue grown under three different environmental conditions. First row: vegetative meristems form short days, 23°C. Second row: transition meristems from long days, 16°C. Third row: inflorescence meristems from long days, 23°C.

To unravel the basic principles underlying the robustness of SAM function by quantitative measurements, we captured SAM domain sizes as well as cell proliferation rates over a wide range of SAM states. To this end we grew *Arabidopsis* plants in three different growth conditions to perturb SAM function and sampled at different developmental stages. We analyzed vegetative meristems of plants grown for 26 days in short days (SD, eight hours of light) under 23°C, meristems during the transition to flowering of plants grown in long days (LD, 23 hours of light) under 16°C and inflorescence meristems of 26 day old flowering plants from a LD, 23°C condition. To quantify the effects of these perturbations, we measured overall SAM size, the size of the functional subdomains, as well as the mitotic index of cells on histological sections of multiple individuals grown under the described conditions. We used *in situ* hybridization of *CLV3* and *WUS* to visualize the SC pool and the OC, respectively. We also monitored the proliferation zone by *in situ* hybridizations of *SHOOT MERISTEMLESS (STM)*, while we used *HISTONE H4* RNA expression as a marker to asses the mitotic index of the CZ and the PZ (see Figure 2).

**Figure 3:**
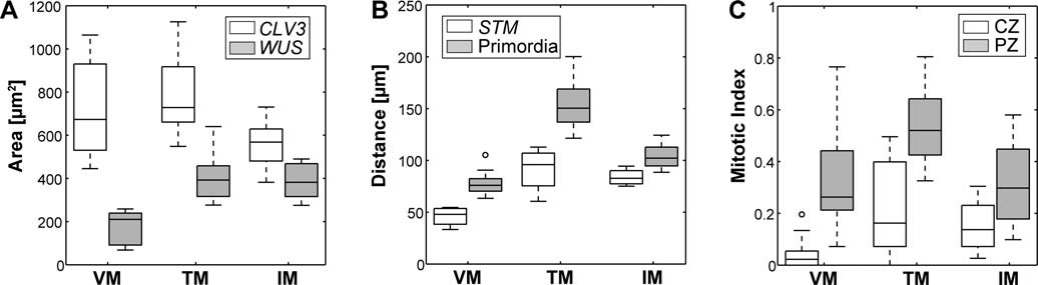
Quantification of SAM markers. Box-plot representation of data derived by image analysis from SAM measurements and *in situ* hybridizations with *CLV3*, *WUS*, *STM* and *HISTONE H4* RNA probes. A) Area of the *CLV3* and *WUS* expression domain. B) Width of the *STM* expression domain and distance between opposing primordia both measured along the apex surface (see Figure 1A). C) Mitotic index (MI) of the CZ and the PZ. Mean and standard deviation of the data is given in Table 1. VM: vegetative meristem from short days, 23°C; TM: transition meristem from long days, 16°C; IM: inflorescence meristem from long days, 23°.

The expression domains of the marker genes were quantified by automated image analysis of individual SAM sections (see Methods for details). The expression domains of *CLV3* and *WUS* could be identified unambiguously and thus the area of their expression could be quantified precisely. In contrast, the expression of *STM* was not restricted to the SAM and extended into the vasculature in all three conditions investigated (see Figure 2). Therefore, the *STM* expression domain in the SAM could not be analyzed in two dimensions, but rather was quantified by its width measured along the surface of the SAM (see Figure 1). To measure the mitotic index, the relative expression area of *HISTONE H4* mRNA in the CZ and the PZ was determined (see Figure 3, Table 1 and Methods).

**Table 1:** Mean and standard deviation of the expression area of *CLV3* and *WUS* and the width of the *STM* expression domain.

	Vegetative meristems (SD 23°C)	Transition meristems (LD 16°C)	Inflorescence meristems (LD 23°C)
***CLV3*** ** [µ** ***m*** **^2^]**	726±206 (14)	790±216 (5)	570±110 (11)
***WUS*** ** [µ** ***m*** **^2^]**	180±80 (6)	409±120 (9)	387±83 (10)
***STM*** ** width [µ** ***m*** **]**	46±10 (4)	91±22 (4)	84±8 (4)
**P-P distance [µ** ***m*** **]**	77±9 (21)	153±24 (14)	104±10 (21)
**Mitotic index CZ**	0.04±0.06 (13)	0.22±0.19 (7)	0.15±0.12 (4)
**Mitotic index PZ**	0.31±0.17 (13)	0.55±0.14 (7)	0.32±0.17 (4)

P-P distance is measured between opposing primordia along the outer SAM surface. The mitotic index is determined as described in the Methods. Sample numbers are given in brackets.

While the overall structure of the SAM remained largely unchanged under all conditions, our quantitative analysis showed that meristem size as measured by the surface distance between opposing primordia varied greatly (see Figure 3 and Table 1). Transition apices on average were twice the size of vegetative meristems and the increase in primordia distance was correlated with a doubling in the width of the of *STM* expression domain (see Figure 3). This expansion of the proliferating cell pool could be viewed as an adaptation to a higher demand for cells during floral transition. Consistent with the enlarged meristem, we also found a two-fold increase in the size of the OC (see Table 1). Surprisingly, the size of the SC domain did not change significantly (Wilcoxon rank sum test for equal medians), pointing to a dynamic and independent regulation of SAM domain sizes. This observation was further supported by the data obtained from inflorescence meristems. Here we found that while meristem size was intermediate between vegetative and transition apices, *STM* and *WUS* domains were practically identical to those in transition apices. Remarkably, we found an almost complementary behavior of the SC domain: While the change in size of the *CLV3* signal was minor and not significant between vegetative and transition apices, despite the dramatic increase in meristem size, the SC domain was reduced in inflorescence apices, which show an intermediate size. This reduction was, however, also not statistically significant (*p* = 0.052). Since the inflorescence meristem is the most mature stage of the apex, this reduction might indicate a gradual loss of stem cells over time. Taken together, our results highlight four important properties of the SAM: (i) Meristem size is highly variable under different growth conditions. (ii) SC number does not strictly correlate with meristem size. (iii) There is no apparent correlation between the size of the OC and the SC domains in different growth conditions. (iiii) The size of the CZ and PZ are not correlated in different growth conditions. It should be noted, however, that our results do not rule out the possibility that the sizes of meristematic domains are correlated under constant conditions.

To extent our analysis beyond meristem organization, we measured the mitotic index of cells as a proxy for cell behavior in the three domains of the SAM. To this end, we quantified cells expressing the S-phase marker *HISTONE H4* by means of *in situ* hybridization. As expected, we detected a significantly higher mitotic index for cells of the PZ when compared to CZ cells in vegetative and transition meristems, consistent with the function of the PZ as cell amplification zone. We also observed on average a two-fold difference in mitotic index between CZ and PZ of inflorescence meristems. However, this difference was not statistically significant, due to the variability of our data. Our results are consistent with previous studies of the cell division pattern in inflorescence meristems, which report a significantly lower number of cell division in the CZ compared with the PZ of the SAM [14], [15]. However, recent studies based on real time lineage analysis of cell division patterns in the L1 have revealed a wide range of cell cycle length distributions in the inflorescence meristem of an individual plant, which might explain the variability of our data [16].

Since the size of the meristem varied over all conditions analyzed, we asked whether cell proliferation rates are also different between the conditions. We found that the mitotic index of CZ and PZ changed significantly between vegetative and transition apices (CZ: *p*≤0.05; PZ: *p*≤0.01; Wilcoxon rank sum test for equal medians). This change was correlated with a significant increase of OC size (*p*≤0.001), the width of the *STM* expression domain (*p*≤0.05) and the overall apex size (*p*≤0.001). Compared to transition apices, inflorescence meristems showed a significantly reduced cell proliferation for the PZ (*p*≤0.01), which was accompanied by a significant decrease in the surface distance between newly emerging primordia (*p*≤0.001).

Taken together, our experimental results provided evidence for a dynamic regulation of meristem domains, which is correlated with a modulation in cell behavior. Over the growth conditions examined, meristem size, as well as the dimensions of the *STM* domain and the OC were correlated with the mitotic index of cells. In contrast, the size of stem cell pool was less correlated with meristem size, despite the fact that also cells of the CZ showed variation in proliferation activity. Since current models based on the *CLV-WUS* feedback hypothesis [13] address meristem maintenance in fixed developmental conditions and therefore cannot account for such a behavior, we developed a quantitative model to uncover the underlying logic of plant stem cell control.

### A quantitative model for the dynamic behavior of meristem cells

To elucidate the underlying principles of meristem and domain size regulation, we developed a quantitative model to describe cell behavior in the shoot apical meristem. Our model is based on the assumption that cell proliferation, cell differentiation and re-specification are the basic size-determining mechanisms in the SAM. In this context, we defined loss of SC identity as differentiation. Two well established interactions between the SC domain and the OC justify our basic assumptions: (i) *CLV3* expression in the SCs is induced by *WUS*, which is expressed in the OC [5]. Since we used both genes as cell pool markers, we accounted for this positive interaction by requiring that SC formation is induced by the OC. Live-imaging experiments revealed that this induction can occur via a fast re-specification of peripheral cell identity to SC identity [8]. We used a linear relationship for the convenience of parameter estimation, which also is a good approximation in case of low *WUS* levels. However, at high levels of *WUS* the re-specification rate probably saturates. (ii) Non-cell autonomous *CLV3* signaling negatively acts on the expression of *WUS* [4], [5], [7], [8]. We accounted for this observation within our model by a negative effect of the SCs on the size of the OC. Thus, SCs increase the differentiation rate of OC cells into non-meristematic cells. Finally, we assumed that the cell proliferation rate is proportional to cell pool sizes. Since we only had an indirect measure for the size of the proliferation zone, we could not use these data for model parameter estimation. Therefore, we only modeled SC and OC. As our data did not allow to reliably distinguish the proliferation rates of OC and the SC pool we used an average proliferation rate for cells in the center of the SAM.

The assumptions listed above were incorporated into the following model for the SC pool size (*S*) and the size of the OC (*O*):
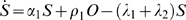
(1)

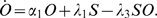
(2)


Figure 1B shows a graphical representation of the model. The cell proliferation rate constant is *α*_1_. The re-specification rate of proliferating cells into SCs depends linearly on the size of the OC with a rate constant *ρ*_1_ accounting for interaction (i). For simplicity, it was assumed that this interaction does not depend on the size of the proliferating cell pool P, which is omitted from the model. The SC differentiation rates are all proportional to *S* with constants *λ*_1_ for the SC-to-OC differentiation and *λ*_2_ for the SC-to-PZ differentiation. The differentiation rate of the OC depends on *S* reflecting interaction (ii) and has a rate constant *λ*_3_. For the purpose of our study we focused our analysis of Equations (1–2) on the steady state size of the SC and OC pools. Equations (1–2) have a trivial steady state at (S^*^, O^*^) = (0,0) which is unstable and a stable, non-trivial steady state given by:
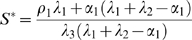
(3)

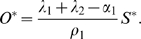
(4)


For biological relevance the steady state must be positive which requires *λ*_1_+*λ*_2_>*α*_1_. In order to yield a predictive model we determined the value of all five model parameters from data. The cell proliferation rate *α*_1_ was calculated using the mitotic index measurements. Note that the value of *α*_1_ depends on the growth condition as if it were controlled by external factors that change under each condition, e.g., nutrient availability or plant hormone levels. All other parameters are assumed to be independent of growth conditions. We use these minimal assumptions, since a specific functional connection between the differentiation and re-specification rates and growth conditions is presently unknown. The re-specification rate *ρ*_1_ was estimated from the experimental data given in [8]. With these values at hand, the steady state Equations (3–4) were used to estimate the differentiation parameters *λ*_1_, *λ*_2_ and *λ*_3_ from the data given in Table 1. The estimated parameter values are listed in Table 2 and Table 3. A detailed description of the parameter estimation can be found in the Materials and Methods. Since the data of cell pool sizes showed a high variability in each condition the parameter values could only be determined up to a certain confidence, which is also given in Table 3.

**Table 2:** Mean and standard deviation of the re-specification rate *ρ*_1_ and the cell proliferation rate of the CZ (*α*_1_) and of the PZ (*α*_2_) in the three investigated conditions.

Parameter	Value (mean±std)
*ρ*_1_	0.1359±0.0577
*α*_1_ (SD 23°C)	0.0042±0.0205
*α*_1_ (LD 16°C)	0.0217±0.0466
*α*_1_ (LD 23°C)	0.0151±0.0389
*α*_2_ (SD 23°C)	0.0315±0.0165
*α*_2_ (LD 16°C)	0.0550±0.0134
*α*_2_ (LD 23°C)	0.0316±0.0179

Parameter *ρ*_1_ was estimated from the data in [8] and *α*_1_ and *α*_2_ were estimated from our *STM in situ* data as described in the Methods. Standard deviations were calculated by error propagation.

**Table 3:** Median and 95% confidence interval for differentiation rates of the three alternative models.

Parameter	Model Equations (1–2)	Model Equations (5–6)	Model Equations (9–10)
*λ*_1_	0.0022 (−0.0022/+0.0341)	0.0082 (−0.0048/+0.0114)	0.0013 (−0.0013/+0.0070)
*λ*_2_	0.0728 (−0.0507/+0.0176)	0.0522 (−0.0204/+0.0218)	0.1418 (−0.0170/+0.0203)
*λ*_3_	0.0180 (−0.0045/+0.0901)	0.0330 (−0.0116/+0.0334)	0.0284 (−0.0079/+0.0228)

Median and confidence intervals were determined by bootstrapping of the data (see Methods). The *χ* ^2^ value of the basic model Equations (1–2) is 3.9651, of the SC-based feedback model Equations (5–6) is 2.6574, and of the OC-based feedback model Equations (9–10) is 1.6162.

**Figure 4:**
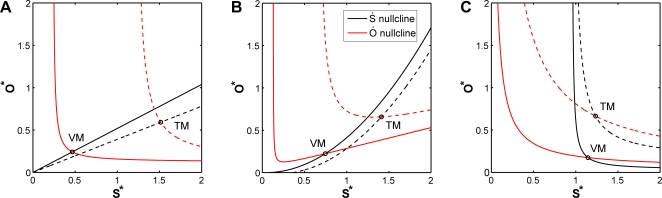
Phase-plane diagrams of alternative models for the SC and OC pool sizes. A) Basic model Equations (1–2). B) SC-based feedback model Equations (5–6), C) OC-based feedback model Equations (9–10). All graphs show the 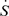 and 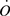 null clines for two different values of the cell proliferation rate *α*_1_. Solid curves: *α*_1_ = 0.0042, as in CZ of vegetative meristems (VM). Dashed curves: *α*_1_ = 0.0217, as in the CZ of transition meristems (TM). The intersection of each pair of null clines corresponds with a steady state (*S^ ˙^*,*O^ ˙^*) and is indicated by a black circle and the corresponding meristem type. Note that the increase in the steady state size of the SC pool (S^*^) due to an increase in *α*_1_ is smaller in both feedback models (B–C) compared to the basic model in A).

Our data showed that the size of the OC and the SC domains varied with the proliferation rate of cells in the CZ. However, the extent of this variation was quite different for both cell pools. Using the optimized model parameters we compared the experimental results with the predicted response of our model to changes in the cell proliferation rate *α*_1_. In the model, the steady state level of the SC pool S^*^ varied by more than a factor of two between the vegetative and the transition meristem using the respective values for the cell proliferation rate (see Figure 4A). In contrast, the steady state level of the OC changed only 1.5 fold. More generally, it can be shown that for any positive parameter values of the model Equations (1–2) the steady state S^*^ is more sensitive to changes in *α*_1_ than the steady state of the OC. This was done by comparing in the relative sensitivities of the steady states S^*^ and O^*^ to a change in *α*_1_.
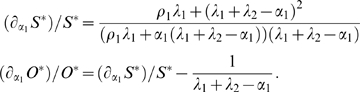



As mentioned above, a positive steady state of S^*^ and O^*^ requires *λ*_1_+*λ*_2_>*α*_1_ and therefore 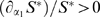 holds. It follows that 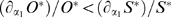. Thus, the change in size of the SC pool due to changes in the cell proliferation rate is larger compared to the respective change in size of the OC. The increased sensitivity of the SC pool is a result of the OC-controlled cell re-specification at the periphery of the SC pool. Our analysis highlights an important prediction of our model: variations in cell proliferation rates as observed in different developmental and environmental conditions lead to changes in SC number. This prediction is supported by our experimental data as transition meristems on average show the largest SC domain compared to vegetative and floral meristems. However, these changes were small compared to those observed for other domains, suggesting that additional mechanisms buffer SC pool size. Noteworthy, this SC variation does not rule out SC homeostasis under constant growth conditions and in fact the model predicts a stable SC number if the cell proliferation rate does not change.

### The role of feedback on the stem cell homeostasis in different growth conditions

SC behavior has mainly been discussed in the context of constant developmental and environmental conditions where the simple negative feedback between SC and OC domains ia able to produce SC homeostasis [1]–[3]. However, our modeling revealed that this feedback alone is unable to buffer SC pool size under changing growth conditions, a behavior, which we had observed experimentally. Thus, additional regulatory mechanisms are necessary to achieve a stabilization of SC number. Alternatively, the large spread of our data might have obscured a more pronounced change in SC pool size. Since we could not distinguish these possibilities experimentally, we investigated them by mathematical modeling asking which additional feedback mechanisms could give rise to SC homeostasis under changing growth conditions. An obvious mechanism to balance the SC pool size is the adaptation of the SC differentiation rate in response to a change in the cell proliferation rate. A similar mechanism was suggested to operate in the SC niche of the colonic crypt [17]. Here, the non-linear regulation of SC differentiation also leads to a robust control of the cell pool size. Following this idea, we introduced two alternative mechanisms that lead to an adaptation of the SC differentiation rate in the SAM. In both cases, the adaptation is based on a secreted differentiation signal *X* that is either produced by the SC pool (i) or by the OC (ii) and is degraded linearly everywhere in the SAM. For simplicity, we assumed that the mobility and decay of *X* are fast compared with the dynamics of the cell pools. Under these conditions the global concentration of *X* is proportional to the size of the cell pool it originates from. Thus, the first model includes a differentiation signal *X*, which is produced by the SC pool. Using the above mentioned approximation *X*∼*S*, lead to a quadratic SC differentiation term.
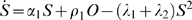
(5)

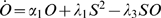
(6)Solving for the non-trivial stable steady state gave,
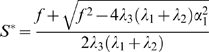
(7)where *f* = *α*_1_(*λ*_1_+*λ*_2_+*λ*_3_)+*ρ*_1_ *λ*_1_ and
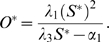
(8)Biological relevance requires 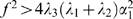 and *λ*_3_ S^*^>*α*_1_. In the alternative model the differentiation signal *X* is produced by the OC, i.e., *X*∼*O*, which can be expressed as:
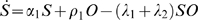
(9)

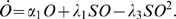
(10)


Note that in order to maintain the SC pool size it is necessary that not only the SC differentiation rate but also the differentiation rate of the OC is regulated by this differentiation signal. The new model also has a trivial steady state (S^*^,O^*^) = (0,0), which is unstable and two alternative non-trivial steady states of which the stable one is:
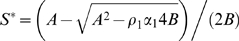
(11)

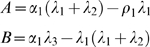


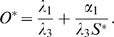
(12)


Here, biological relevance requires *A*>0 and *A* ^2^>*ρ*_1_ *α*_1_4*B*. The differentiation rates of each model were estimated as described above and are given in Table 3.

The results for the SC-based feedback mechanism are shown in the phase plane diagram of Figure 4B. A five-fold increase in the cell proliferation rate increases the size of the OC three-fold and that of the SC pool two-fold. Thus, mechanism (i) reduces the sensitivity of the SC pool size to changes in the cell proliferation rate compared with the basic model in Figure 4A. The reduction in sensitivity is even stronger for the alternative OC-based feedback mechanism as can be seen in Figure 4C. Since the size of the OC increases in response to an elevated cell proliferation, the differentiation signal and thus the SC differentiation rate regulated by the OC increases accordingly, leading to a stabilization of the SC pool size. Thus, mechanism (ii) allows for an almost perfect SC homeostasis in the various SAM states. However, the reduced sensitivity to variations in the cell proliferation rate is accompanied by a high sensitivity towards changes in other model parameters. E.g. a 10% increase in *λ*_3_ or *ρ*_1_ leads to a loss of a stable steady state. Therefore, an OC-based feedback mechanism is much more fragile compared with the SC-based feedback mechanism. Taken together, both suggested mechanisms lead to a reduction in the sensitivity of the SC pool size to changes in cell proliferation rate. If the differentiation signal originates from the OC, an almost perfect SC homeostasis under different environmental and developmental conditions could be achieved. This robustness, however, is accompanied by fragility in other model parameters.

### Regulation of cell output generated by the stem cells of the shoot apical meristem

**Figure 5:**
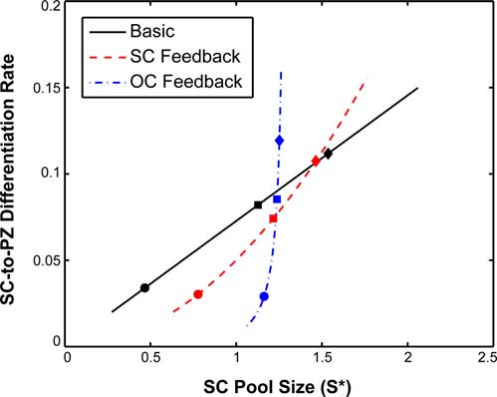
Dependence of the CZ cell output rate and SC pool size. The output rate is defined by the fraction of SCs that differentiate into PZ cells per unit time. Note that while the basic model Equations (1–2) show a linear increase in SZ size with, both models including a feedback on the SZ differentiation rate exhibit a reduced (Equations (5–6)) or almost absent (Equations (9–10)) change in the SC pool size while delivering the same increase in cell output rate. Circles: vegetative meristem from short days, 23°C. Diamonds: transition meristem from long days, 16°C. Squares: Inflorescence meristem from long days, 23°C.

The control of overall cell production per time, or cell output rate, is the major task for the shoot apical meristem to serve its function to supply the growing plant with an appropriate amount of building material. To address the question of how much the SC pool contributes to the varying amount of cells produced in the SAM, we asked how the size of the SC pool in the different conditions is correlated with the differentiation rate into PZ cells using our three fitted models. Figure 5 shows the cell output rate of the CZ in dependence of the SC pool size. Each pair of values was calculated by varying *α*_1_ continuously between 0.001 and 0.03. Noteworthy, all three models predict a higher SC output rate in transition meristems when compared to vegetative meristems. Thus, an increase in SC proliferation contributes to meeting a higher demand for cells during floral transition. While the increase in the cell output rate is almost the same for all three models, the change in SC pool size is not. While for the basic model Equations (1–2) the SC size scales linearly with cell output rate, both models including additional feedback mechanism show a reduced change in SZ size. For the OC-based feedback, the SC pool size is almost independent of the output rate.

### Prediction of *CLV3* and *WUS* over-expression phenotypes

**Figure 6:**
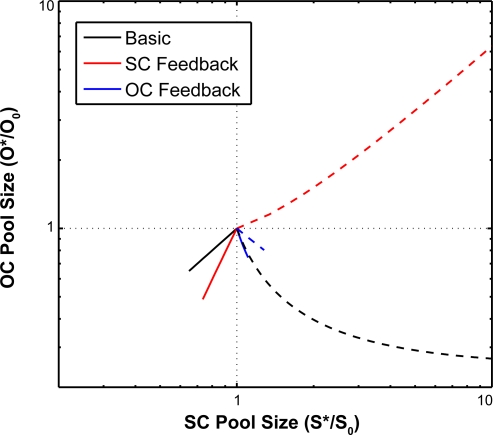
Cell pool sizes in response to changes in *CLV3* or *WUS* activity. The responses to elevated *CLV3* signaling are indicated by solid lines, the effect of ectopic *WUS* expression are denoted by dashed lines. All domain sizes are given relative to the WT pool sizes denoted by *S*_0_ (SC) and *O*_0_ (OC), respectively. Note that only the basic model Equations (5–6) can recover the experimental phenotypes. Here, enhanced *CLV3* signaling, simulated by increasing *λ*_3_, leads to a reduced OC and SC pool size. Ectopic *WUS* over-expression, simulated by including a constant SC production rate, enlarges the SC domain and leads to a decrease of the endogenous *WUS* activity. Black: basic model Equations (5–6). Red: SC-based feedback model Equations (9–10). Blue: OC-based feedback model Equations (9–10).

Since all fitted models can explain the experimental data with reasonable accuracy (see *χ* ^2^ values, caption of Table 3), we asked whether they could also correctly predict the results of experimental modulations in *CLV3* and *WUS* expression [4], [5], [18]. Since our modeling approach did not allow us to exactly replicate the experimental setup of published experiments, such as those from Müller et al. or Schoof and colleagues [5], [18], we tested two alternatives. First, we increased SC number as a means to simulate an ectopic expression of *WUS*, while as a second approach, we increased the differentiation rate of OC cells, to account for an increased *CLV3* signaling emanating from the same number of stem cells as described by Müller et al. [18]. Elevated *CLV3* levels are known to repress endogenous *WUS* expression, despite the fact that variations in *CLV3* signaling are compensated over a wide range [18]. Thus, we expected that an artificial enlargement of the SC domain, as in plants ectopically expressing *WUS* [5], would reduce OC size. Conversely, an increase in the negative SC-to-OC signaling should lead to a reduction in both OC and SC size. Using these phenotypes as a test case, we varied SC production and OC differentiation rates in all our three models in order to simulate the respective over-expression experiments. Figure 6 shows a comparison of the phenotypes predicted by our three models. In order to avoid a statement based on unknown and therefore arbitrary over-expression rates, only the functional relations between the sizes of the SC pool and the OC are shown. Interestingly, only the basic model without additional feedback is in agreement with the experimentally observed phenotypes. Here, the SC domain size increases in response to ectopic *WUS* expression, while the OC size, which is a proxy for the endogenous activity of the *WUS* promoter, is decreased, but not shut down completely (see Figure 6, black dashed curve). Elevated *CLV3* levels lead to a reduction of the SC and OC size as expected from experiments (see Figure 6, black solid curve). In contrast, the SC-based feedback model correctly predicts only the response to increased *CLV3* levels (Figure 6 solid red line), but fails to do so in the case of ectopic *WUS* expression. In this scenario the SC-feedback model predicts an increase in OC size, which is in disagreement with experimental observations (see Figure 6, red dashed curve). The OC-based feedback model is very sensitive to experimental perturbations and allows only very limited ectopic *WUS* over-expression as well as elevation of *CLV3* levels. The response in the later scenario is also mispredicted (see Figure 6, blue solid curve).

Thus, both feedback mechanisms invoked to buffer the variations in SC number under changing growth conditions interfere with the ability of the model to explain modulations of the system at the genetic level.

## Discussion

The complexity of known and unknown regulatory interactions in the SAM precludes an intuitive understanding of plant stem cell control [19]. It is reasonable to expect that the quantitative regulation of cell number is dependent on a feedback system that can sense the number of SC and adjust SC proliferation and differentiation rates accordingly. Therefore, we expect that these rates ultimately depend on the number of cells in the SAM. Adopting this view allowed us to circumvent the problems of extracting quantitative information from the numerous known genetic interactions. Instead, we choose a cell pool size dependent description of the SC regulation. One advantage of this approach is that we were able to directly address the question of how the output rate of meristem cells without stem cell property (referred to as differentiated cells) is regulated, which is an important property of meristem function. It is noteworthy that despite this abstraction our model is still mechanistic in the sense that it can be used to predict the effect of genetic or environmental perturbations that change cell proliferation or differentiation rates or otherwise change the size of a specific cell pool in the SAM.

Based on this idea we have quantified different cell pool sizes under various environmental and developmental conditions, which cause an adaptation of the SAM organization. We were able to observe a systematic adaptation of cell pool sizes and cell proliferation rates of the CZ and PZ of the SAM in different conditions. While the variations in meristem and subdomain size as well as cell proliferation rates were striking, the correlation between these responses was non-trivial. Thus, we have employed mathematical modeling to deduce rules of meristem behavior from our experimental data. We have formulated a model based on the known domain structure of the SAM and determined the unknown cell differentiation rates by fitting the model to our new experimental data. This formed the basis for a systematic study of the influence of cell proliferation on the cell pool sizes of the SAM. An important simplification of our modeling approach is the implicit treatment of the spatial structure of the SAM by using cell pools that are connected via differentiation rates. This simplification allows us to arrive at a coarse-grained but nevertheless quantitative picture of SC regulation since all model parameters were identifiable from our data. While a cell-based model would allow answering specific questions, e.g., about the regulation of cell differentiation at the pool boundaries, it would also require a much finer spatial and temporal resolution of the data to identify all its parameters. With the advance of live imaging techniques [20], [21] it will become possible to study cell pool dynamics in the SAM with much greater detail and thus allow quantitative modeling of cell behavior with high spatial and temporal resolution.

The most important observation made from our experimental data is that the meristem is a highly plastic tissue, which undergoes substantial changes in domain organization and cell behavior in response to environmental and developmental cues. In the context of this plasticity, the low variation in SC number under the growth conditions tested is remarkable. While our dataset is too limited to draw final conclusions, it suggests that under changing growth conditions, SC number is well buffered but not in perfect homeostasis, which is compatible with a homeostatic SC behavior under constant conditions. Since a simple feedback model is unable to account for this observed stability of SC number, we have included additional feedback systems into our model. A thorough analysis of these three models shed new light onto the dynamical constraints of SC regulation in the SAM: none of the models was able to correctly predict *CLV3* and *WUS* over-expression phenotypes and SC homeostasis under changing growth conditions at the same time. One explanation for this could be that, due to data limitation, some of the underlying assumptions derived from experimentation might be incorrect. Alternatively, there could be unknown regulatory connections between the feedback systems, which are able to modulate the responses. However, the adaptation to changes in the environment involves the fully functional regulatory system, while interference at the genetic level, such as in over-expression experiments, might disable some parts of the regulatory network. Thus, we believe that the results obtained from modeling meristem behavior under various growth conditions are more relevant than those aimed at explaining over-expression phenotypes.

As a central assumption of our study we treated the cell proliferation rate as an externally controlled quantity that is adapted during the different environmental and developmental conditions. This allowed us to derive a quantitative model of the central meristem zone, which is able to predict the effect of experimental perturbations. However, additional internal feedback mechanisms might operate in the SAM to control SC proliferation and differentiation. For example, it was shown that ectopic co-expression of *WUS* and *STM* not only induces ectopic SCs, but also leads to organ formation, i.e., differentiated tissue from SCs [22]–[24]. Consistently, WUS is a direct activator of the floral patterning gene *AGAMOUS* [25], demonstrating its involvement in both proliferation and differentiation. Plant hormones strongly contribute to the regulation of this balance and in the context of the root meristem the phytohormone cytokinin was shown to play an important role in cell differentiation [26]. Conversely, cytokinin is an essential signal for cell proliferation in the SAM [27]–[29]. A direct link between stem cell control and cytokinin signaling came from the finding that *WUS* directly represses the expression of *ARABIDOPSIS RESPONSE REGULATOR 7 (ARR7)*, a negative element of cytokinin signal transduction [30]. Interestingly, *ARR7* has a negative effect on *WUS* expression, providing another layer of feedback regulation. The intricate spatial regulation of cell proliferation and differentiation within the meristem almost certainly involves modulation of the cell cycle machinery in the various SAM domains. It has recently been shown that *CYCLIN DEPENDENT KINASES* of the B2 class (*CDKB2;1 and CDKB2;2*) are not only essential for proper cell cycle progression, but also for the correct spatial organization of the SAM [31]. Interestingly, the expression of these genes is dependent on *WUS* and *STM* function and their activity is at least partially mediated by plant hormones, such as auxin and cytokinin. Thus, the adaptation of cell proliferation leading to different cell pool sizes in different environmental and developmental conditions could be the result of a complex and highly branched regulatory network. Our study is a first attempt to uncover the basic regulatory principles of this network by a combined approach of quantitative data collection and modeling.

## Materials and Methods

### Plant Material and Growth Conditions

Plants of Columbia (Col-0) background were grown under three different light and temperature conditions in order to elicit variations in SAM size. All plants were harvested after 26 days. The three growth conditions were: short day (SD), 23°C = vegetative SAM; long day (LD), 16°C = SAM during floral transition; LD, 23°C = inflorescence SAM.

### *In situ* hybridization

*In situ* hybridizations were performed using a standard protocol [30]. The goal was the precise quantification of marker expression area and not the absolute or relative expression level. Therefore, in order to achieve high optical resolution of the stained tissue and avoid spreading of the NBT-BCIP dye, the staining reaction was stopped when single cells gave a clear signal. The maximum staining length was one day.

### Image Acquisition and Image Analysis

Images were taken with a Zeiss AxioCam HR camera mounted to a Zeiss Axioplan 2 microscope and taken with a resolution of 0.54 µ*m* ^2^ per pixel. All images were acquired with the Zeiss AxioVision Image software and saved in TIFF format. Subsequent image analysis was performed on the intensities of the red channel, which gave the sharpest staining signal of the three color channels represented in the TIFF images. The expression area of *CLV3*, *WUS* and *HISTONE H4* was determined by thresholding relative to the intensity of the unstained tissue of the same image. This image specific thresholding allows corrections for sample and image specific properties, such as background intensity and illumination. The threshold was determined by the mean of the unstained tissue intensity minus four standard deviations. Thresholded pixels, which did not correspond to cell-shaped areas with a diameter ≥3 µ*m* were removed. The mitotic index was determined as the ratio of the thresholded area to the total area of a quadratic selection. For each developmental condition the size of total area of selection for the CZ was adapted to the mean size of the *CLV3* expression area under this condition. The selection of the PZ was directly adjacent to either side of the CZ and had the same size. The mitotic index of the PZ was averaged over both sides. The distance between the two inner primordia was measured along the outer epidermal layer of the SAM. All image analysis was performed with the Imaging Toolbox of the MATLAB software from Math Works, Inc. All MATLAB scripts are available from the authors upon request.

### Parameter Estimation and Mathematical Modeling

Numerical analysis of the model was performed with the MATLAB software from MathWorks, Inc. For parameter estimation the measured areas *A* for *CLV3* and *WUS* were transformed to volumes *V* assuming a spherical symmetry of the cell pools:
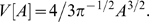



This data transformation is appropriate in order to reflect the three dimensional structure of the cell pools in the SAM. However, we want to note that the main conclusions of the modeling are not dependent on this data transformation. Subsequently, all volumes were scaled to relative quantities by taking the mean *CLV3* expression volume of the inflorescence meristem as a reference volume *V_ref_*. The transformed data relate to the dynamical variables of our model as follows: SC pool size *S_i_*∼*V_ref_*_,*i*_/*V_ref_*, size of the OC *O_i_*∼*V_ref_*_,*i*_/*V_ref_*, where *i* is the index of the sampled conditions.

The work of Reddy et al. revealed that cell re-specification precedes cell proliferation and is probably controlled by the OC [8]. This justifies the simple model *S* ^˙^ = *ρ*_1_ *O* to calculated the re-specification rate *ρ*_1_ via the approximation:
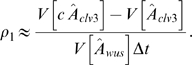

*Aˆ* *_clv_*_3_ and *Aˆ* *_wus_* are the mean *CLV3* and *WUS* expression areas of the inflorescence meristem respectively. The factor *c* is the fold increase in *CLV3* expression area after a time Δ*t* = 24 h and is in the order of two [8]. The standard error of *ρ*_1_ is calculated by error propagation (see Table 2).

The mitotic index of a given tissue corresponds to the probability of observing a proliferating ( = stained) cell within the tissue and is given by the ratio of the expression length of the *HISTONE H4* marker (*L_H_*_4_) to the total cell cycle length (*L_cc_*). The average cell proliferation rate *α* of a given cell pool can then be calculated as:
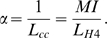



We assume *L_H_*_4_ = 10 h as the average *HISTONE H4* marker expression length [32]. The calculated values of the CZ and PZ are given in Table 2.

The remaining differentiation rates 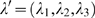 were estimated by least square fitting of the steady state equations of our models to the mean of the *CLV3* and *WUS* expression areas in all three developmental stages of the SAM minimizing the functional:
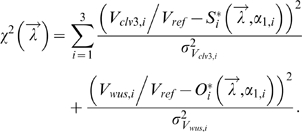



Confidence intervals for the differentiation rates were determined by a bootstrap procedure. One thousand bootstrap samples were generated from the complete data and the mean and standard deviation of each sample was used for parameter estimation. The resulting parameter distributions were used to calculate the median and 95% confidence interval of the three differentiation rates (see Table 3).

### Simulation of elevated *CLV3* levels and ectopic *WUS* over-expression

In order to simulate the effect of elevated *CLV3* levels, the differentiation parameter λ_3_ was increased from its basal level. Thereby, the SC pool size can be used as a proxy for the activity of the endogenous *CLV3* promoter. Ectopic *WUS* over-expression was simulated adding a constant production rate to the dynamical equation for the SC pool size. This enables to visualize the activity of the endogenous *WUS* promoter by monitoring OC levels. The new steady states were computed by numerical integration.
